# Identification of Filovirus Entry Inhibitors from Marine Fungus-Derived Indole Alkaloids

**DOI:** 10.3390/md23010023

**Published:** 2025-01-03

**Authors:** Leah Liu Wang, Javier Seravalli, Brett Eaton, Yi Liu, Michael R. Holbrook, Wen-Jian Lan, Shi-Hua Xiang

**Affiliations:** 1 Nebraska Center for Virology, School of Veterinary Medicine and Biomedical Sciences, University of Nebraska-Lincoln, Lincoln, NE 68583, USA; 2 Department of Biochemistry, University of Nebraska-Lincoln, Lincoln, NE 68588, USA; 3 Integrated Research Facility at Fort Detrick, National Institute of Allergy and Infectious Diseases, Frederick, MD 21702, USA; 4 Holland Computing Center, University of Nebraska-Lincoln, Lincoln, NE 68588, USA; 5 School of Pharmaceutical Sciences, Sun Yat-sen University, Guangzhou 510006, China

**Keywords:** indole alkaloids, filovirus, entry inhibitors, marine fungus, *Fusarium* sp., secondary metabolites, therapeutic agents

## Abstract

Filoviruses, mainly consisting of the two genera of *Ebolavirus* and *Marburgvirus*, are enveloped negative-strand RNA viruses that can infect humans to cause severe hemorrhagic fevers and outbreaks with high mortality rates. However, we still do not have effective medicines for treating these diseases. To search for effective drugs, we have identified three marine indole alkaloids that exhibit potent activities against filovirus infection. Thus, it is suggested that marine indole alkaloids can be a valuable compound source for filovirus drug screening and development. Since marine indole alkaloids comprise a large diverse group of secondary metabolites, their biological properties would be helpful for pharmaceutical drug development to treat various filovirus infections.

## 1. Introduction

Filoviruses are a group of viruses that have a filamentous form and have been classified as the family *Filoviridae* [1,2,3]. To date, there are five recognized genera and two of them are the well-known *Ebolavirus* and *Marburgvirus* [2]. The *Ebolavirus* includes five species, *Zaire Ebola virus* (EBOV), *Sudan virus* (SUDV), *Bundibugyo virus* (BDBV) and *Taï Forest virus* (TAFV). The *Marburgvirus* includes only two species, *Marburg virus* (MARV) and *Ravn virus* (RAVV) [2]. EBOV and MARV are the well-known filoviruses since they have caused human severe hemorrhagic fever and deadly outbreaks with high mortality rates, as high as 90% (WHO website) [4,5,6]. Although a limited vaccine and two antibody-based treatments against Ebola were recently approved in December 2019 and 2020, respectively (as per announcements on the FDA website), there are still no small molecule-based drugs that have been approved for treating filovirus infections [7,8]. Therefore, it is essential to develop therapeutics against filovirus infection and to potentially avoid epidemic.

Filoviruses are enveloped single-stranded negative-sense RNA viruses with a genomic size of approximately 19 kb [1,4]. The envelope spike glycoprotein (GP) is the only viral protein on the virion surface. The active state has a structure consisting of three GP molecules that are required for viral entry [9,10,11]. The GP is biosynthesized as a single polypeptide but later cleaved by the cellular protease Furin into the GP1 and GP2 subunits, but the subunits are still connected through an inter-subunit disulfide bond [12]. The entry of filoviruses such as Ebola occurs through micropinocytosis and transportation via trafficking into early and late endosomes [13,14,15]. In the late endosomes, GP1 is further cleaved by Cathepsin proteases in order to remove the glycan cap and also the heavily glycosylated mucin domain, which exposes the receptor binding site (RBS) that interacts with the endosomal receptor Nieman-pick C1 (NPC1) [9,16,17]. The NPC1 functions as a transporter of lysosomal cholesterol but it is utilized by filoviruses for entry into the endosomes [18,19,20,21]. The cleaved GP (GPcl), also called primed GP, includes the cleaved GP1 and part of the GP2; it is able to bind the cellular receptor NPC1 [22,23]. The binding of viral GPcl to the cellular receptor NPC1 triggers a GP2 conformational change that initiates the membrane fusion process. The GP2 fusion loop exposure and insertion into the cell membrane creates a pre-hairpin type of structure that leads to the formation of a 6-helix bundle [24,25,26], which results in viral and cellular membrane fusion; that way, the viral genome can reach the cell cytoplasm for replication [14,27,28,29,30,31]. Thus, receptor binding is a key step for viral entry. Therefore, an effective way to prevent viral infection is by blocking the receptor binding.

Marine organism-derived natural compounds, such as indole alkaloids, are a valuable source for pharmaceutical development. Marine-derived indole alkaloids have been extensively studied in recent years [32,33]. Their structural diversity provides a wide opportunity for the development of therapeutic agents in various applications. They have been found to be useful as antiviral, antimicrobial and anticancer [33] agents. We have previously reported that indole alkaloids from marine-derived *Fusanrium* sp. showed activity against mammalian cell Zika virus infection [34]. To further study these compounds, we have identified three indole alkaloids in this report that have strong activities against filovirus infections. It is suggested that marine-derived indole alkaloids would be a valuable source for antiviral drug development.

## 2. Results

### 2.1. Screening of Indole Alkaloid Compounds

**Figure 1:**
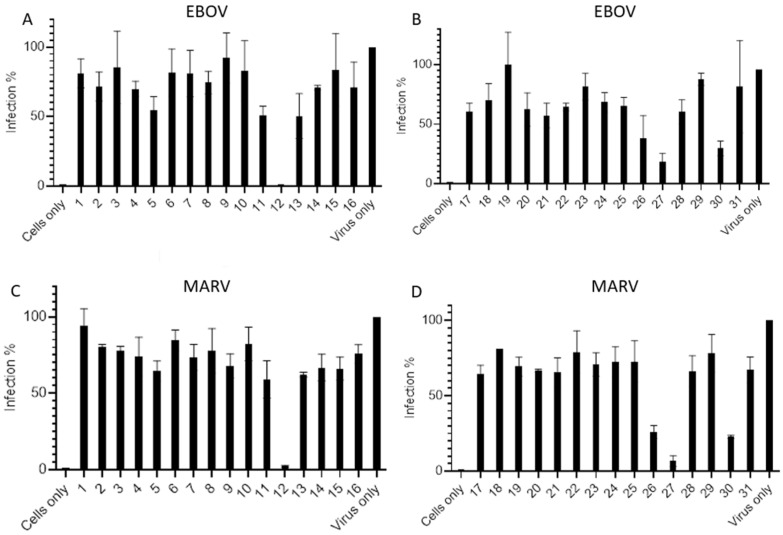
Screening of thirty-one W compounds at 10 µM concentration against pseudotyped filoviruses. EBOV: (**A**,**B**): MARV: (**C**,**D**). Cells only, TZM-bl cells without compounds as the negative control; Virus only, only viruses without compounds as the positive control.

Thirty-one indole alkaloid compounds, isolated from marine-derived fungi, were applied to our pseudotyped virus platform for neuralization assay against Ebola and Marburg virus infections. The compound concentration was first set up at 10 µM for activity screening. Three compounds (W12, W26 and W27) were found to have modest activities against both Ebola and Marburg viruses (Figure 1).

### 2.2. Cytotoxicity Assay of Indole Alkaloid Compounds

**Figure 2:**
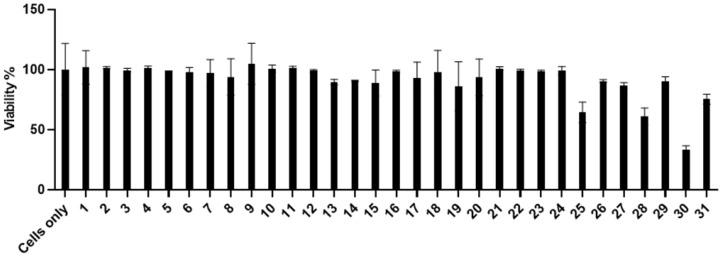
Evaluation of compound cytotoxicity in TZM-bl cells using MTT assay. All thirty-one W compounds were applied for the test at 10 µM concentration for the test Cells only, without compounds.

**Figure 3:**
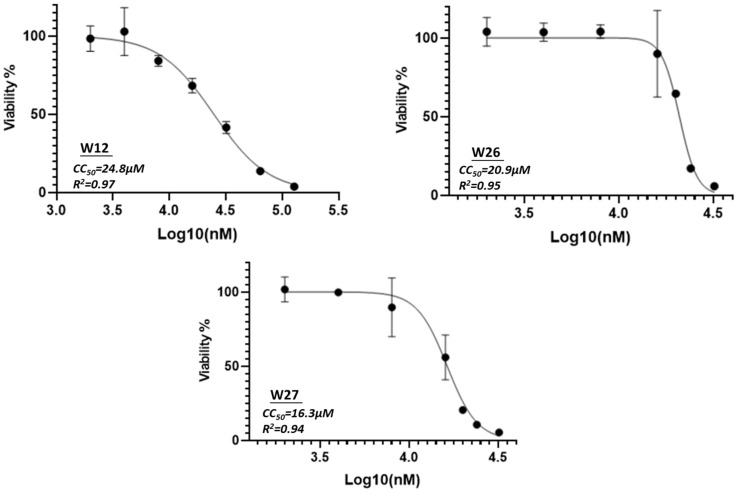
CC_50_ analysis (50% cytotoxicity concentration) of W12, W26 and W27 in TZM-bl cells using MTT assay. The toxicity was evaluated from 2 µM to 32 µM of compound concentrations.

The compound cytotoxicity was also evaluated at the concentration of 10 µM using a cell viability assay (MTT); the results are shown in Figure 2. Most compounds did not show significant cytotoxicity except compounds W25, W28 and W30, especially compound W30, which showed the highest cytotoxicity (CC_50_ value). Based on the cytotoxicity data and the activity data, we have further evaluated three compounds (W12, W26 and W27) for CC_50_ (50% cytotoxicity concentration) analyses. The CC_50_ values are 24.8 µM, 20.9 µM and 16.3 µM for compound W12, W26 and W27, respectively (Figure 3).

### 2.3. Inhibition Assay (IC_50_) of Indole Alkaloid Compounds

**Figure 4:**
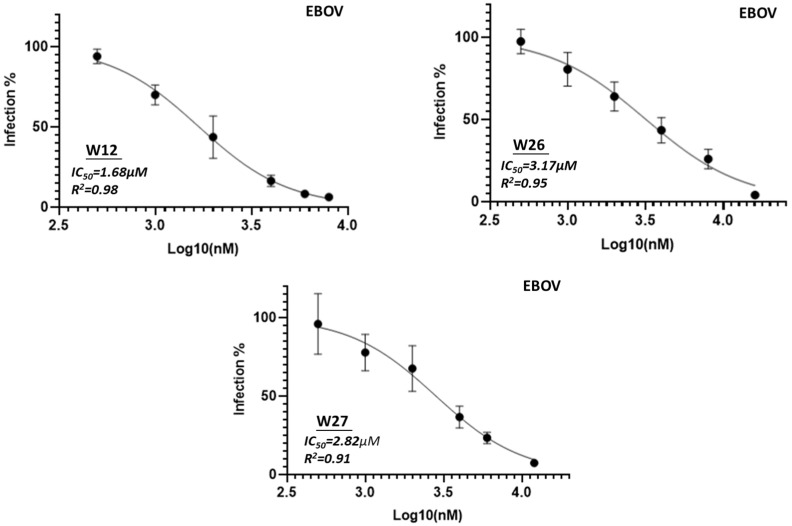
IC_50_ analysis (50% maximal inhibitory concentration) of compound W12, W26 and W27 against pseudotyped virus EBOV. Serial 2-fold dilutions of compound from 0.5 µM to 32 µM were evaluated.

**Figure 5:**
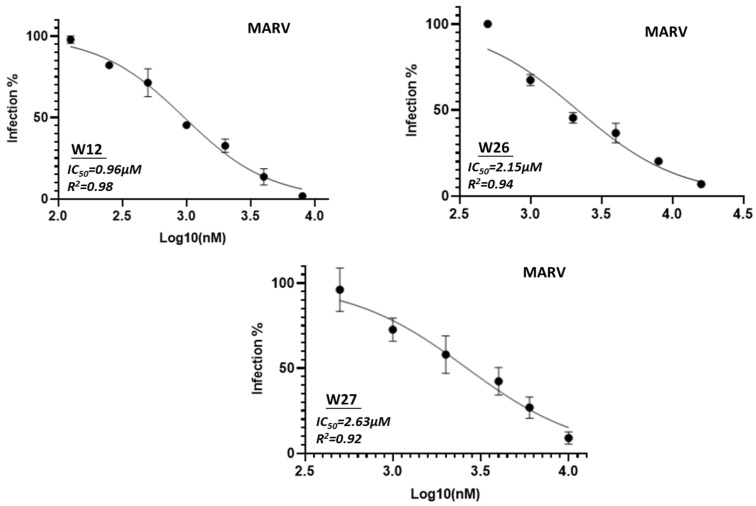
IC_50_ analysis of compound W12, W26 and W27 against pseudotyped MARV. Serial 2-fold dilutions of compound from 0.5 µM to 32 µM were evaluated.

The three compounds W12, W26 and W27 were selected for further evaluation for IC_50_ assay (50% maximal inhibitory concentration) against pseudotyped filoviruses, which were performed at the BSL-2 laboratory. Since the HIV-based pseudotyping method for Ebola has been well documented [35,36,37,38], we used this method to prepare pseudotyped EBOV and MARV. These pseudotyped viruses were evaluated using the TZM-bl cells by which the inhibition activity can be easily evaluated by detecting the luciferase activity in a luminometer. These three compounds showed activity against Ebola virus with IC_50_ values around 1.68 µM, 3.17 µM and 2.82 µM, respectively (Figure 4). For Marburg virus, the viruses are slightly more potent with IC_50_ values of 0.96 µM, 2.15 µM and 2.63 µM, respectively (Figure 5).

### 2.4. Inhibition Assay (IC_50_) of Indole Alkaloid Compounds Against Infectious Filoviruses

**Figure 6:**
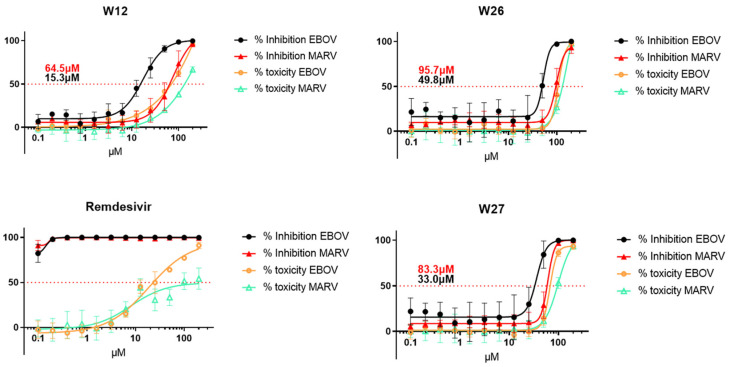
IC_50_ analysis of compound W12, W26 and W27 against infectious virus EBOV and MARV in human Huh7 cells. Compound concentrations from 0.1 µM to 100 µM were evaluated. The toxicity was also evaluated from 0.1 µM to 100 µM of compound concentrations. Remdesivir was used as positive controls.

To validate the inhibition data from pseudotyped viruses, we evaluated the three selected alkaloids against infections by the Ebola and Marburg viruses, in the BSL-4 containment suite. In this experiment, human Huh7 cells were used, and the compound toxicity was evaluated simultaneously with the activity tests. Remdesivir was used as a positive control. The IC_50_ values of W12, W26 and W27 were 15.3 µM, 49.8 µM and 33.0 µM for EBOV and 64.5 µM, 95.7 µM and 83.3 µM for MARV, respectively (Figure 6). In general, the three compounds exhibited more activity against EBOV than against MARV, but the IC_50_ values are higher than those measured with pseudotyped viruses. These differences may come from such conditions as the use of different cells.

### 2.5. Inhibition Specificity Analysis

**Figure 7:**
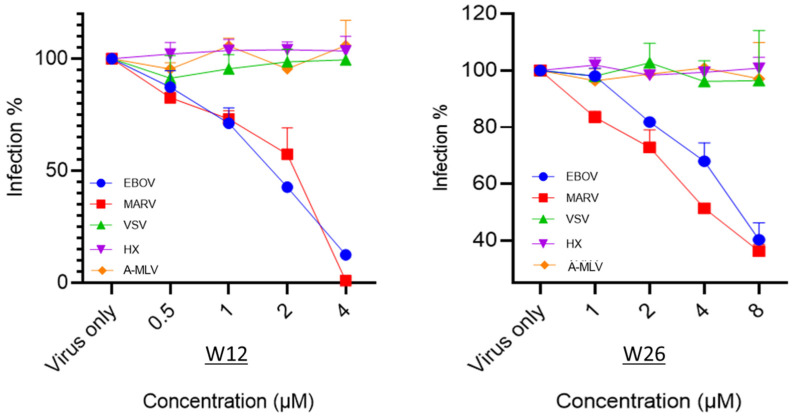
Specificity testing of compound W12 and W26 against different pseudotyped viruses (EBOV, MARV, HIV, VSV and A-MLV). HIV strain, HXBc2 (HX); vesicular stomatitis virus (VSV); amphitropic murine leukemia virus (A-MLV).

To evaluate the specificity of these compounds, we tested other commonly used RNA viruses to assess whether they were affected by the indole alkaloids. The three viruses were the following: (1). Vesicular stomatitis virus (VSV), which is an enveloped, negative-sense RNA virus that infects a wide variety of mammalian cells. (2). Amphitropic murine leukemia virus (A-MLV), a commonly used retrovirus that infects a wide range of cell types from many distinct species. (3). Human immunodeficiency virus (HIV), a retrovirus that infects CD4-positive cells. Two of the compounds, W12 and W26, were applied for testing and the results are shown in Figure 7. The results clearly indicate that while the two indole alkaloid compounds W12 and W26 were neutralizing the filoviruses EBOV and MARV, neither of them did so for the other three viruses (Figure 7). It appears then that these compounds have the specificity for inhibiting filovirus infection.

### 2.6. Molecular Docking and Structural Analysis

**Figure 8:**
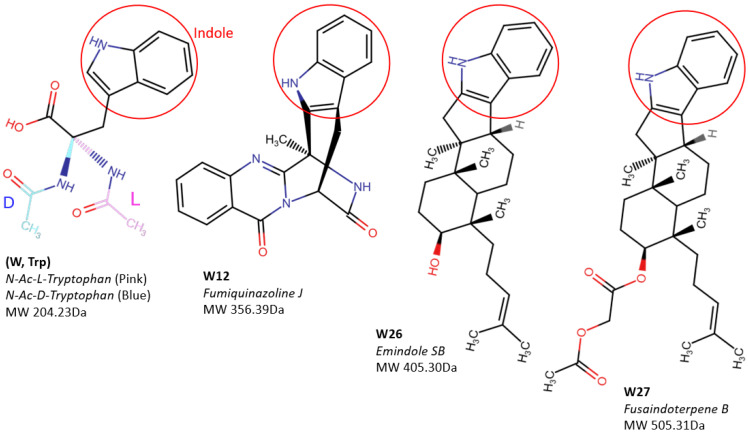
Structural comparisons of compounds W12 (*Fumiquinazoline J*), W26 (*Emindole SB*) and W27 (*Fusaindoterpene B*) with the amino acid *Tryptophan* (Trp)-derived *N-Acetyl-L-Tryptophan* (Ac-L-Trp) and *N-Acetyl-D-Tryptophan* (Ac-D-Trp) forms. The common indole ring is in a red circle and the differences in their side groups are shown. All molecule weights (MWs) of these compounds are also shown below their structures.

**Figure 9:**
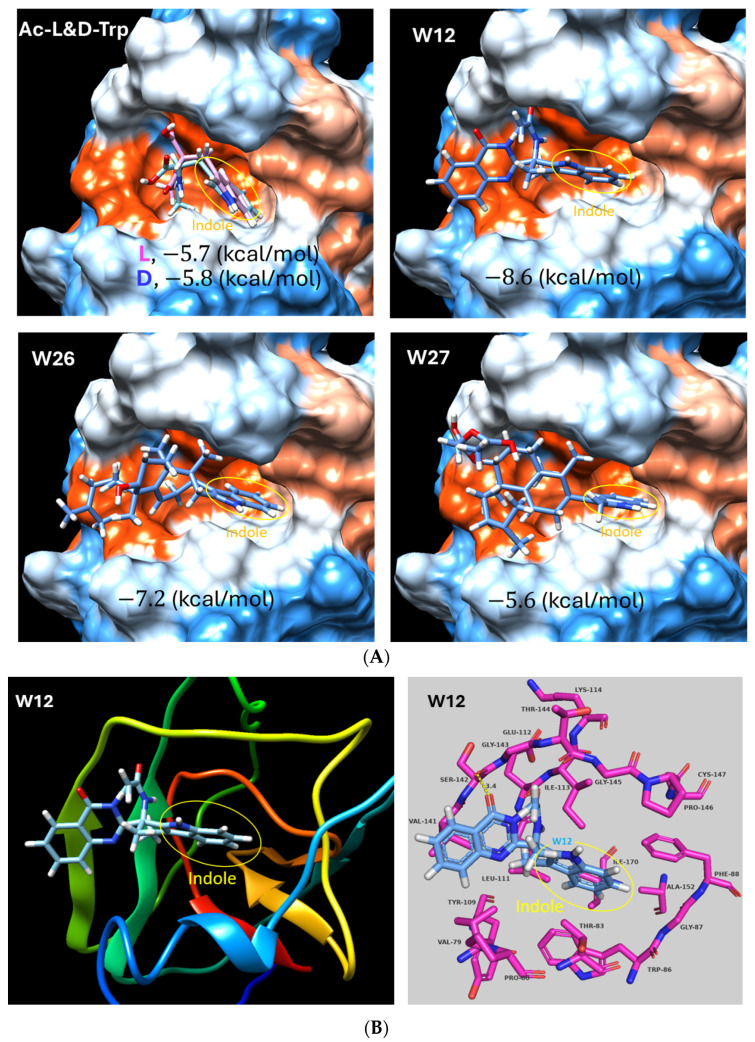
(**A**). Molecular docking of compounds W12, W26, W27and the amino acid Tryptophan (Trp)-derived *N-Acetyl-L-Tryptophan* (Ac-L-Trp) and *N-Acetyl-D-Tryptophan* (Ac-D-Trp) forms in the NPC1 receptor binding site of EBOV using AutoDock and illustrations using the Chimera (Surface models). (**B**). Compound W12 ribbon model (**left**) and the interactions model ((**right**), made using PyMol). All the docking scores (kcal/mol) are labeled in the models. Indole ring is in the yellow circle. Surface colors: red, hydrophobic; blue, hydrophilic.

The structures of the three compounds (W12, W26 and W27) all contain an indole ring that may be able to interact with the receptor NPC1 binding site (Figure 8). To test this hypothesis, we conducted a molecular docking analysis using the AutoDock Vina program. The data suggest that these three compounds did have affinities to the receptor binding site with docking score values of −8.6, −7.2 and −5.6 (kcal/mol), respectively (Figure 9A). Interestingly, the indole ring was inserted into the deep cavity of the binding site. We also tried to assess the binding of the amino acid Tryptophan, which also contains the indole ring. Both N-Acetyl-L- or D-Tryptophan (Ac-L- or D-Trp) showed docking into the RBS and also showed some activity against pseudotyped Ebola viruses (Figure 9A and Table 1). The compound W12 has the best docking score among these compounds, which appears to match its inhibition activities. From the interactions of compound W12 with the residues within the RBS, there are mostly hydrophobic interactions with the residues LEU-111, ILE-113, VAL-141 and ILE-170. One close electrostatic interaction is with residue SER-142 at a distance of 3.4 Å. It is suggested that hydrophobic interaction is the major binding force in this RBS.

### 2.7. Compound Binding to the Receptor Binding Domain (RBD)

**Figure 10:**
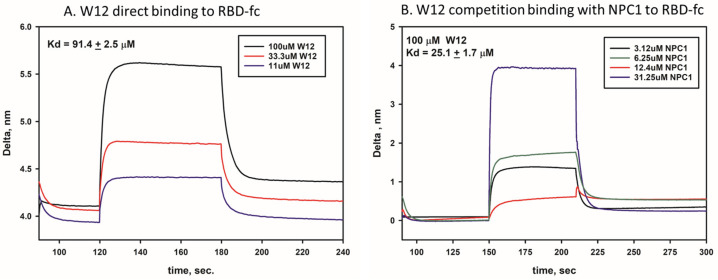
Compound binding to EBOV GP-RBD. Compound W12 binding affinity at pH 6.1 was evaluated using biolayer interferometry (BLI) assay method. (**A**). W12 direct binding to RBD of EBOV-GP. (**B**). W12 binding competition with receptor NPC1 to RBD of EBOV-GP.

To demonstrate compound binding, we have conducted a protein-based binding assay using the biolayer interferometry assay (BLI). A construct in which the RBD protein of EBOV-GP was fused to the Fc portion of a human IgG was used in conjunction with anti-human IgG Fc Capture (AHC2) biosensors for the detection of compound W12 binding to the RBD protein. The binding assays were conducted at pH 6.1, which is a mild acidic condition that is observed in late endosomes, and which is required for viral entry into mammalian cells. Compound W12 was bound to the RBD of EBOV-GP with a Kd value of 91.4 µM (Figure 10A). To confirm the competitive nature of the binding site, we designed a competition binding assay for the receptor NPC1_C domain. The compound W12 did show competition binding with the NPC1_C domain to the EBOV GP-RBD with an observed Kd value of 25.1 µM when 100 µM of compound W12 was used (Figure 10B). In contrast, binding of the NPC1_C domain for RBD alone was found to be 9.6 µM. From these observations, it is suggested then that compound W12 as well as other similar compounds like W26 and W27 bind the RBD receptor in a competitive manner with respect to NPC1, which results in the blocking of the endosomal viral entry.

## 3. Discussion

In the present work, three marine-derived indole alkaloids from Fusarium sp. have been identified to show strong activities against Ebola and Marburg virus infections. It has been noticed that they are more potent for EBOV than for MARV, based on the infectious virus neutralization data. This is most likely due to the sequence differences between the receptor binding sites. The corresponding viral receptors have about ~70% difference in their protein sequences, for EBOV and MARV [39,40,41]. Paradoxically, they share the same intracellular target receptor NPC1 (Niemann-pick C1 receptor) at the endosomes or lysosomes, which is used by the viruses for entry into mammalian cells. This implies that the receptor binding sites (RBDs) are structurally similar [42,43]. The discordance between pseudo-viruses and infectious viruses can be explained by the following reasons (Table 1). Firstly, the cells and viral strains used are different. Pseudovirus inhibitions used TZM-bl cells and Zaire strain (EBOV) and Marburg strain (MARV), but the virus inhibition assays described herein used human Huh7 cells with Angola (EBOV) and Makona (MARV) strains. Secondly, the pseudoviruses are single-round infection viruses that only inhibit viral entry, while the infectious viruses can replicate after entry that may affect the outcomes of inhibition.

**Table 1:** Summary of major W compound inhibition against filoviruses.

Compound	Virus	IC_50_ (µM) Pseudovirus	CC_50_ (µM)	SI ^1^	IC_50_ (µM) Infectious Virus
N-Ac-L-tryptophan	EBOV	>48 *	>60	-	
	MARV				
N-Ac-D-tryptophan	EBOV	>48 *	>60		
	MARV				
W12	EBOV	1.68	24.8	15	15.3
	MARV	0.96		26	64.5
W26	EBOV	3.17	20.9	7	49.8
	MARV	2.15		10	95.7
W27	EBOV	2.28	16.3	7	33.0
	MARV	2.63		6	83.3

^1^ SI, Selectivity index, which was calculated by CC_50_/IC_50_. * The IC_50_ values were estimated based on the preliminary tests.

The compound W12 exhibited the best activities against EBOV and MARV from both platforms of pseudoviruses and infectious viruses. In the docking results, W12 also shows the highest docking score among the indole alkaloid compounds. This correlation of the docking score and functional activity also suggests that the W compounds target the receptor binding site for blocking viral entry.

It is exciting to know that these marine fungus-derived indole alkaloids can be used as entry inhibitors against filovirus infection. To demonstrate whether this phenomenon is a general one for the class of indole alkaloids, we also included the indole-derived amino acid Tryptophan as part of the panel of compounds tested herein. The n-acetyl-L and D-isomers of Tryptophan were assessed. Both showed similar activities (IC_50_ > 48 µM), which indicate that there is no difference from other isomers, but they do have the function of inhibiting Ebola virus infection (Table 1). Thus, the indole ring may play a key role in targeting this receptor binding site. Indeed, the docking results also indicate that the Trp compounds can also be docked in the RBS and show similar postures as the three W compounds in which indole rings are inserted into the deep cavity but have lower scores (Figure 9A).

In recent years hundreds of indole alkaloids from marine fungi have been isolated. Five indole alkaloids from the marine-derived fungus *Fusarium equiseti* LJ-1 have been shown to exhibit anti-cancer cell activities through in vitro tests [44]. Prenylated indole alkaloids obtained from co-cultures of marine-derived fungi *Aspergillus sulphureus* and *Isaria felina* (*Beauveria felina*) have activity inhibiting the colony formation of human prostate cancer cells [45]. Prenylated indole alkaloids from the marine-derived fungi also show activity against two human cancer cell lines, H1975 and HepG-2 [46]. Antifouling indole alkaloids from two marine derived fungi *Penicillium* sp. and *Aspergillus sydowii* were found to have anti-bacterial activity [47]. Indole Alkaloids from Marine Fungus *Pseudallescheria boydii* F44-1 also have anticancer activity [48]. Indole alkaloids isolate of *Penicillium janthinellum* Biourge have activity inhibiting EGF-induced malignant transformation of JB6 P+ Cl 41 cells in a soft agar [49]. Indole Alkaloids from the Fungus *Talaromyces assiutensis* JTY2 exhibit broad spectrum antibacterial activities [50]. Undoubtedly, marine fungi from deep sea environments may produce many unique compounds [51]. The family of marine indole alkaloids from fungi is remarkably diverse and can become a valuable resource for finding natural compound-based medicines for cancer, bacterial and viral diseases. Indeed, these natural compounds can lead to significantly drug design and development for human health [33,52,53,54,55,56,57,58,59,60,61].

In conclusion, three marine-derived indole alkaloids were identified to have strong activities against filovirus infection including both EBOV and MARV. Especially, the compound W12 exhibits more potency and less cytotoxicity than the rest of the members of the panel assayed herein. Therefore, marine-derived indole alkaloids provide a valuable source for drug development against filovirus infection.

## 4. Materials and Methods

### 4.1. Chemicals, Strains and Plasmids

All thirty-one compounds (named W) and see the list in the Supplementary (Table S1) were provided by Dr. Wen-Jian Lan. These compounds were extracted from the marine fungus *Fusarium* sp., which was isolated from the South China Sea. The envelope glycoprotein genes (GPs) synthesized are based on the sequences from the GenBank, Ebola virus (Zaire ebolavirus, accession number: AIO11753.1), Marburg virus (Marburg marburgvirus Uganda 02Uga07, accession number: ACT79201.1). The VSV-G and A-MLV-env genes and the plasmid pSG3ΔEnv were obtained from the NIH AIDS Reagent Program.

### 4.2. Pseudotyping Ebola Viruses

All pseudotyped viruses were made from the HIV-1 backbone plasmid pSG3ΔEnv as the HIV-based pseudotyping of Ebola has been demonstrated [35]. The envelope genes (GPs) of Ebola and Marburg viruses were synthesized and cloned into the pCDNA3.1(+) expression plasmid. Both plasmids of pSG3ΔEnv and the GP envelope were co-transfected into 293T cells in a 10 cm plate using transfection reagent polyethyleneimine (PEI). After incubation at 37 °C for two days, the supernatants were harvested after a short spin to remove cell debris and stored at −80 °C.

### 4.3. Pseudovirus Neutralization Assay

The virus neutralization assay was performed in the BSL-2 laboratory in 96-well plates using pseudotyped Ebola viruses and TZM-bl cells (6000/well) as this cell line was engineered with a luciferase report gene under the inducible promoter of Tat factor. The viral particles and compound samples were mixed and transferred onto the target cell wells for infection. One-day post-infection, the supernatants were removed, the cells were washed once with PBS and incubated in fresh media for one more day. Thereafter, the cells were lysed in 1x Passive Lysis Buffer (Promega, Madison, WI, USA) and kept at room temperature for 20 min for luciferase assay. The luciferase activity was measured using luciferin substrate (Promega) with the Veritas Luminometer. The neutralization activities were calculated by comparing them with the control samples.

### 4.4. Infectious Virus Neutralization Assay

Wild-type EBOV (Makona C07 strain, IRF0192) and MARV (Angola, IRF0202) were used for compound neutralization assays, which were conducted in the BSL-4 containment at the Integrated Research Facility of NIH at Fort Detrick, Frederick, MD, USA. Human Huh7 cells were set seed 6000 cells/well 30 µL in 10% FBS medium in a standard plate and infected with 0.5 MOI for EBOV in a 48 h assay, but 1.5 MOI for MARV as that assay was only for 24 h. We had also calculated toxicity as a product of how many nuclei remained relative to infected, untreated control. The toxicity at each drug concentration was determined by cell loss compared to the infected control. We simply counted Hoechst-stained nuclei and compared them. There were 4 replicates per plate at each condition plus 24 infected, untreated controls and 24 uninfected, untreated controls. The IC_50_ was determined by using nonlinear regression in GraphPad Prism software, version 10.0.

### 4.5. Cell Viability Assay

The cell viability was measured by the standard MTT assay. TZM-bl cells (3000/well) were seeded in a 96-well plate and incubated for 24 h at 37 °C. The medium was removed and replaced with 100 μL of compound sample in complete DMEM for two days and then themedium was replaced with 100 μL complete DMEM for continuingly culturing for one more day. The medium was removed, and the cells were washed once with PBS for analysis. The 50 μL solution of 5 mg/mL MTT [3-4, 5-dimethylthiazol-2-yl)-2, 5-diphenyltetrazolium bromide] (Sigma-Aldrich, St. Louis, MO, USA) was added to each well. The plates were incubated for 3 h at 37 °C, and the absorbance was measured at 570 nm and with 650 nm (background) wavelength.

### 4.6. Molecular Docking

Molecular docking was performed using the AutoDock Vina program [62,63], an open-source tool for efficient and effective ligand–receptor docking. The AutoDock Vina computes a docking score that approximates the standard chemical potentials between the receptor and ligand [63]. The docking score empirically weights superficially physics-based terms (e.g., the 6–12 van der Waals interactions and Coulomb energies) to account for the difference between energies and free energies. While the docking score does not directly measure binding affinity, it provides a relative estimate of ligand–receptor interaction strength within the limitations of the scoring model.

Both the receptor and ligands were preprocessed for docking. The receptor used for docking was the Ebola-GP obtained from Protein Data Bank, PDB ID 5F1B [23]. The preparation of the receptor involved removing the GP2 portion and cutting off the GP1 regions of Arg31-Val65 and Val181-GLN188, the addition of hydrogens, and removal of water molecules using Chimera [64]. The grid box for the active site was carefully defined based on the EBOV GP-RBD (the GP1 region from Gly67 toVal180) allowing for efficient and sufficient exploration of the docking poses. The preparation of the molecular structures of ligands (i.e., N-Ac-L-tryptophan, N-Ac-D-tryptophan, W12, W26 and W27) involved adding hydrogen atoms to stabilize the bonds for accurate docking prediction.

After docking, the structural analysis was conducted using Chimera and PyMol (Schrödinger, Inc., New York, NY, USA). Chimera was used to visualize the docking results, providing a detailed inspection of the docking poses. PyMOL was subsequently employed for advanced visualization to analyze molecular interactions.

### 4.7. Biolayer Interference

Biolayer interference assays were conducted as described by the biosensor manufacturer (Sartorius AG, Göttingen, Germany). The biosensors consisted of anti-human IgG Fc Capture conjugates that are used for the kinetic and equilibrium characterization of IgG or IgG fusion proteins. The RBD-Fc fusion protein was obtained from Sino Biological. The assay consisted of 5 steps: 30 s buffer baseline with 20 mM MES pH = 6.1, 60 s RBD loading, 60 s dissociation baseline, 60 s ligand association and 90 s ligand dissociation. The dissociation steps used the same buffer. The volumes for the association steps were 4 µL, while the dissociation steps were 250 µL. The BLI binding isotherms were analyzed with the software provided by the manufacturer (Sartorius AG, Göttingen, Germany). Graphs were generated with Sigmaplot 14.0 (Grafiti LLC, Palo Alto, CA, USA).

### 4.8. Data Statistical Analysis

GraphPad Prism software (version 10.0, San Diego, CA, USA) was used to determine average values, standard errors and IC_50_. The statistical method used to calculate IC50 was nonlinear regression. All data were from three experiments and each sample was in triplicate for pseudotyped virus neutralization assay.

## Data Availability

The original data presented in the study are included in the article/Supplementary Materials; further inquiries can be directed to the corresponding author.

## Supplementary Materials

The following supporting information can be downloaded at: https://www.mdpi.com/article/10.3390/md23010023/s1, Table S1: List of indole alkaloid compounds (W) tested.
