# Supplementary material for: Identification of Filovirus Entry Inhibitors from Marine Fungus-Derived Indole Alkaloids
Source: Mar Drugs. 2025 Jan 3;23(1):23. doi: 10.3390/md23010023 (PMC11766795; doi:10.3390/md23010023)
Supplement: Supplementary file 1 [file marinedrugs-23-00023-s001.zip › Table_S1_CompoundList-20250112.pdf]

Table_S1 List of Indole Alkaloids Compounds (W or WJL) Tested*

W (orWJL) number	Structure	Formula	Molecular Weight (Da)	Source/Notes
1	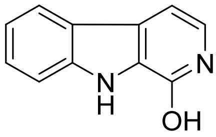	$C_{11}H_8N_2O$	184.20	*Frontiers in Microbiology*, 2022, 13, 947226.
2	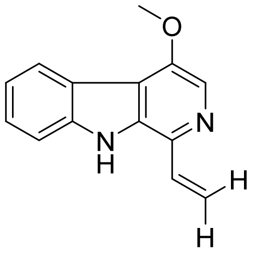	$C_{14}H_{12}N_2O$	224.26	Mar. Drugs 2025, 23(1)
3	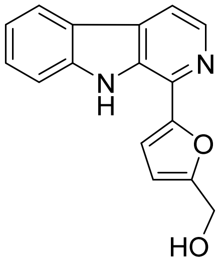	$C_{16}H_{12}N_2O_2$	264.28	*Bioorganic Chemistry,* 2021, 116, 105375.
4	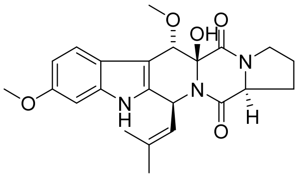	$C_{23}H_{27}N_3O_5$	425.48	*Marine drugs* **2025**, *23*(1), 4; *https://doi.org/10.3390/md23010004*
5	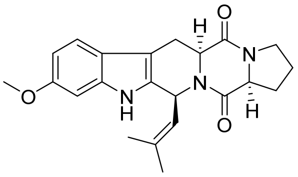	$C_{22}H_{25}N_3O_3$	379.45	*Marine drugs* **2025**, *23*(1), 4; *https://doi.org/10.3390/md23010004*
6	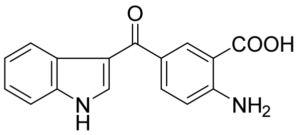	$C_{16}H_{12}N_2O_3$	280.00	*Marine drugs* **2025**, *23*(1), 4; *https://doi.org/10.3390/md23010004*

7	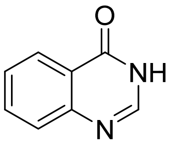	$C_8H_6N_2O$	146.15	Mar. Drugs 2025, 23(1)
8	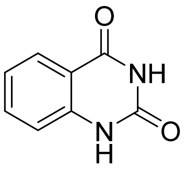	$C_8H_6N_2O_2$	162.15	Mar. Drugs 2025, 23(1)
9	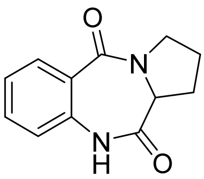	$C_{12}H_{12}N_2O_2$	216.24	*Marine drugs* **2025**, *23*(1), 4; *https://doi.org/10.3390/md23010004*
10	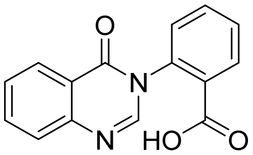	$C_{15}H_{10}N_2O_3$	266.26	Mar. Drugs 2025, 23(1)
11	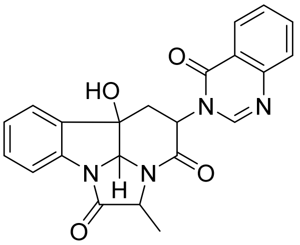	$C_{22}H_{18}N_4O_4$	402.40	Mar. Drugs 2025, 23(1)
12	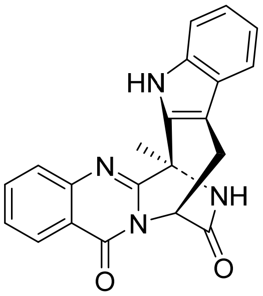	$C_{21}H_{16}N_4O_2$	356.39	*Organic Letters, 2017, 19(18), 4888-4891*
13	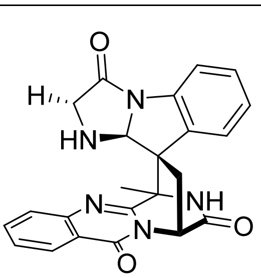	$C_{23}H_{19}N_5O_3$	413.43	Mar. Drugs 2025, 23(1)

14	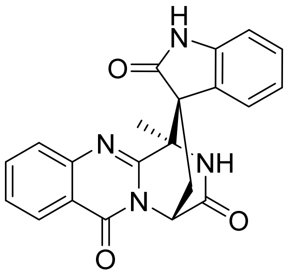	$C_{21}H_{16}N_4O_3$	372.38	*Marine drugs* **2025**, *23*(1), 4; *https://doi.org/10.3390/md23010004*
15	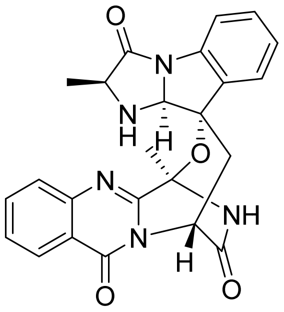	$C_{24}H_{21}N_5O_4$	443.46	*Marine drugs* **2025**, *23*(1), 4; *https://doi.org/10.3390/md23010004*
16	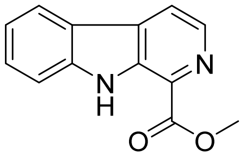	$C_{13}H_{10}N_2O_2$	226.24	*Fitoterapia*, 2023, 166, 105433.
17	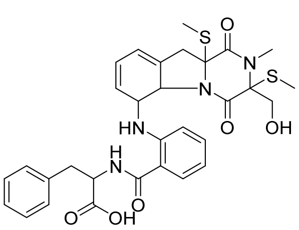	$C_{31}H_{34}N_4O_6S_2$	622.76	*Marine drugs* **2025**, *23*(1), 4; *https://doi.org/10.3390/md23010004*
18	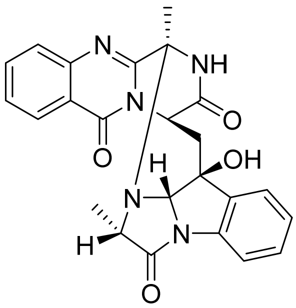	$C_{23}H_{19}N_5O_4$	443.46	*Marine drugs* **2025**, *23*(1), 4; *https://doi.org/10.3390/md23010004*
19	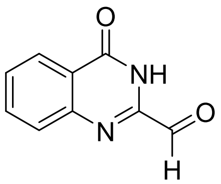	$C_9H_6N_2O_2$	174.16	Mar. Drugs 2025, 23(1)
20	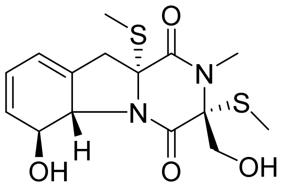	$C_9H_8N_2O_4S_2$	356.46	*Bioorganic Chemistry*, 2021, 116, 105375.

21	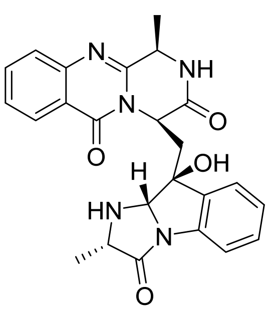	$C_{24}H_{23}N_5O_4$	445.48	Mar. Drugs 2025, 23(1)
22	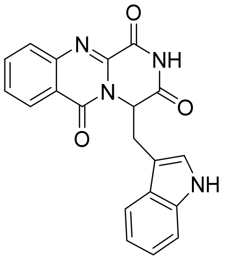	$C_{20}H_{14}N_4O_3$	358.11	*Bioorganic Chemistry,* 2021, 116, 105375.
23	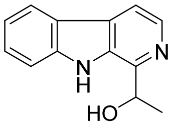	$C_{13}H_{12}N_2O$	212.09	*Frontiers in Microbiology*, 2022, 13, 947226.
24	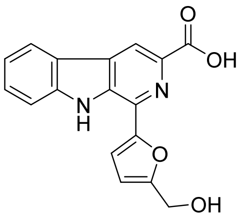	$C_{17}H_{12}N_2O_4$	308.08	*Bioorganic Chemistry,* 2021, 116, 105375.
25	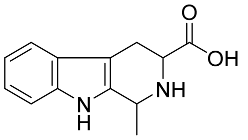	$C_{13}H_{14}N_2O_2$	230.11	*Frontiers in Microbiology*, 2022, 13, 947226.
26	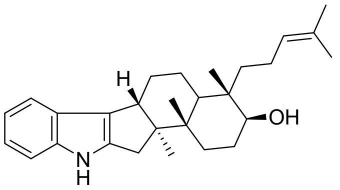	$C_{28}H_{39}NO$	405.30	*Journal of Natural Products*, 2020, 83(11), 3372-3380.
27	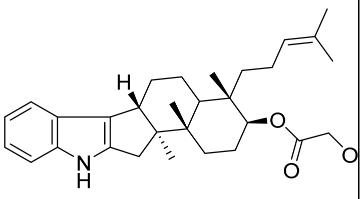	$C_{32}H_{343}NO_4$	505.31	*Journal of Natural Products*, 2020, 83(11), 3372-3380.
28	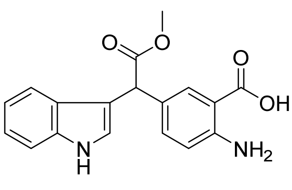	$C_{18}H_{16}N_2O_4$	324.11	*Marine drugs* **2025**, *23*(1), 4; *https://doi.org/10.3390/md23010004*

29	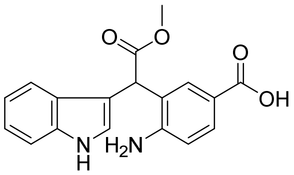	$C_{18}H_{16}N_2O_4$	324.11	*Marine drugs* **2025**, *23*(1), 4; *https://doi.org/10.3390/md23010004*
30	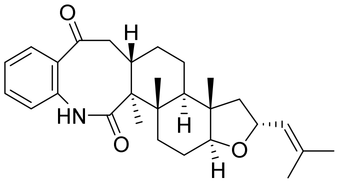	$C_{28}H_{37}N_2O_4$	435.27	*Journal of Natural Products, 2020, 83, 11, 3372–3380.*
31	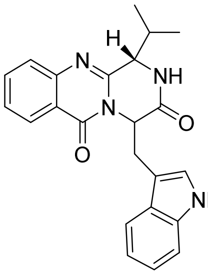	$C_{23}H_{22}N_4O_2$	386.17	*Journal of Natural Products*, 2020, 83, 4, 1082–1091.

**All these compounds were provided by Dr. Wen-Jian Lan.*
